# Emulsome-Based Nanocarrier System for Controlled 4-Phenylbutyric Acid Delivery and Mechanistic Mitigation of Arsenical-Induced Skin Injury via Foam Application

**DOI:** 10.3390/pharmaceutics18010053

**Published:** 2025-12-30

**Authors:** Nethra Viswaroopan, Meheli Ghosh, Sharvari M. Kshirsagar, Jasim Khan, Jennifer Toral-Orduno, Ritesh K. Srivastava, Mohammad Athar, Ajay K. Banga

**Affiliations:** 1 Center for Drug Delivery Research, Department of Pharmaceutical Sciences, College of Pharmacy, Mercer University, Atlanta, GA 30341, USA; nethra.viswaroopan@live.mercer.edu (N.V.); meheli.ghosh@live.mercer.edu (M.G.); sharvari.milind.kshirsagar@live.mercer.edu (S.M.K.); 2 UAB Research Center of Excellence in Arsenicals, Department of Dermatology, University of Alabama at Birmingham, Birmingham, AL 35294, USA; jkhan@uabmc.edu (J.K.); jorduno@uab.edu (J.T.-O.); riteshsrivastava@uabmc.edu (R.K.S.); mathar@uab.edu (M.A.)

**Keywords:** 4-phenylbutyric acid, emulsome, lewisite, decontamination, foam formulation

## Abstract

**Background**: Lewisite, a potent chemical warfare agent, induces rapid and progressive cutaneous damage, necessitating treatment strategies that offer both immediate decontamination and prolonged therapeutic action. This study aimed to develop and evaluate a composite topical formulation comprising 4-phenylbutyric acid (4-PBA)-loaded emulsomes embedded within a foam vehicle to address both aspects of vesicant-induced skin injury intervention. **Methods**: Emulsomes composed of a stearic acid–cholesterol solid lipid core stabilized by a lecithin shell were prepared via thin film hydration and optimized by varying lipid ratios and drug loading parameters. Formulations were characterized for drug loading, particle size, and zeta potential. Physicochemical compatibility was assessed using Fourier transform infrared spectroscopy (FTIR) and differential scanning calorimetry (DSC) analyses. Stability was evaluated under accelerated refrigerated (25 °C/60% RH) and room temperature (40 °C/75% RH) conditions. The optimized formulation was incorporated into a foam base and evaluated for decontamination efficiency, drug release kinetics, in vitro permeation, and in vivo efficacy. **Results**: The selected formulation (E2) exhibited high drug loading (17.01 ± 0.00%), monodisperse particle size (PDI = 0.3 ± 0.07), and stable zeta potential (−40 ± 1.24 mV). FTIR and DSC confirmed successful encapsulation with amorphous drug dispersion. The emulsome-foam demonstrated dual functionality: enhanced decontamination (66.84 ± 1.27%) and sustained release (~30% over 24 h), fitting a Korsmeyer–Peppas model. In vitro permeation showed significantly lower 4-PBA delivery from E2 versus free drug, confirming sustained release, while in vivo studies demonstrated therapeutic efficacy. **Conclusions**: This emulsome-foam system offers a promising platform for topical treatment of vesicant-induced skin injury by enabling both immediate detoxification and prolonged anti-inflammatory drug delivery.

## 1. Introduction

Vesicants are chemical warfare agents that induce severe dermal injury through rapid cellular membrane disruption, reactive oxygen species (ROS) generation, and inflammatory cascade activation, leading to erythema, edema, necrosis, and blister formation [1,2]. Among these, arsenical vesicants like Lewisite (2-chlorovinyldichloroarsine) pose a significant threat due to their low volatility, potent vesicant activity, and high lipophilicity, enabling rapid stratum corneum penetration within seconds [3]. Dermal exposure at doses as low as 3–14 µg/cm^2^ causes visible erythema and blistering, while systemic absorption results in multiorgan toxicity [4,5]. Despite being banned under the Chemical Weapons Convention, legacy munitions and potential misuse necessitate modern field-deployable countermeasures [6,7].

Current post-exposure decontamination relies on rapid skin flushing, which is limited by timing, availability, and lack of therapeutic action against ongoing molecular injury [8,9]. The FDA-approved antidote, British Anti-Lewisite (BAL), chelates trivalent arsenicals but has a narrow therapeutic window, severe side effects, and requires impractical intramuscular injection [8]. Water-soluble analogs like dimercaptosuccinic acid (DMSA) show better safety profiles but reduced efficacy in treating cutaneous Lewisite injury, particularly when applied topically [10]. Critically, Lewisite-induced injury continues progressing hours after exposure through oxidative and inflammatory pathways, even after initial decontamination [11,12], highlighting the need for a topically applicable, dual-function formulation capable of both decontaminating residual agent and delivering local therapy.

Mechanistic studies reveal that Lewisite toxicity involves oxidative stress, endoplasmic reticulum (ER) homeostasis disruption, and unfolded protein response (UPR) activation [13]. Prolonged UPR activation drives CHOP overexpression, misfolded protein accumulation, NF-κB activation, and keratinocyte apoptosis [12,14]. In this context, 4-phenylbutyric acid (4-PBA), an FDA-approved small molecule for urea cycle disorders, has emerged as a promising therapeutic candidate [15]. As a chemical chaperone, 4-PBA enhances protein folding capacity, reduces ER stress, and suppresses inflammation and ROS production [16]. Studies using phenylarsine oxide (PAO), a Lewisite analog, demonstrate that topical 4-PBA significantly reduces histopathological damage, pro-inflammatory cytokine expression, and keratinocyte apoptosis [13]. However, 4-PBA’s physicochemical properties (MW 164.2 g/mol, log P ≈ 2.5, melting point ~50 °C), while favorable for passive dermal diffusion, result in rapid clearance, poor skin retention, and potential irritation without proper formulation [17,18,19].

Multiple formulation strategies have been previously explored to enhance the topical delivery of 4-PBA for the treatment of arsenical-induced skin injuries. Microsponges, polymeric microparticles with highly porous internal networks, previously developed in our lab, have also been used to entrap 4-PBA for sustained release via gel formulations. Although they prolonged drug release profiles, the drug loading capacity in microsponges was significantly low, and the formulation lacked a built-in decontamination function, limiting its applicability in emergency exposure settings [20]. Separately, blank foam formulations were optimized for rapid chemical removal and skin-safe application. However, they did not include a therapeutic payload, making them more suitable as first-line decontaminants rather than complete treatment systems [21]. Foam formulations with 4-PBA-loaded nanoparticles, such as chitosan–TPP nanoparticles incorporated into a foam base, were developed as a dual-function platform aimed at both decontamination and therapeutic delivery. While this system enabled encapsulation of 4-PBA through electrostatic interactions and facilitated moderate skin retention, the overall drug loading remained minimal, and the formulation was susceptible to environmental instability due to pH- and salt-sensitive ionic crosslinking [18]. These observations underscore the need for a formulation combining stable drug encapsulation, improved loading capacity, dermal retention, and integrated decontamination.

To address these limitations, this study employs emulsomes, hybrid lipid-based nanoparticles with a solid lipid core surrounded by phospholipid bilayers, as a nanocarrier platform for topical 4-PBA delivery [22]. This structure combines the high encapsulation efficiency and sustained release of solid lipid nanoparticles with the biocompatibility and flexibility of liposomes [22]. The solid lipid core stabilizes lipophilic drugs while phospholipid shells enhance bioadhesion, facilitate skin penetration, and protect the payload [22,23,24]. Compared to traditional nanocarriers, emulsomes offer greater compositional control, physical stability, longer shelf life, and avoid synthetic polymers that may cause immunogenicity [25,26,27,28]. Their size (100–300 nm), slightly negative zeta potential, and cholesterol-rich phospholipid shells facilitate follicular uptake, epidermal retention, and favorable interaction with stratum corneum lipids [23,29,30,31].

The emulsomes were incorporated into a topical foam formulation (F30) previously developed for skin application [21]. Foams offer ease of spreading, rapid absorption, a non-greasy finish, and can be applied without pressure or friction, which is crucial for burned or blistered skin [32,33]. This hybrid emulsome-in-foam system achieves dual functionality: surfactant components aid in lifting residual chemical agents from skin, while encapsulated 4-PBA is delivered in a controlled manner to target ER stress and inflammatory cascades driving vesicant pathology. Emulsomes were systematically optimized for particle size, zeta potential, polydispersity index, entrapment efficiency, and drug loading, with foam incorporation evaluated for appearance, spreadability, stability, and release performance. This work presents a novel nanocarrier-based approach that combines chemical decontamination and targeted therapy in a single formulation, addressing a critical gap in countermeasure development for first responders, military personnel, and civilians at risk of arsenical exposure.

## 2. Materials and Methods

### 2.1. Materials

For this study, 4-PBA, stearic acid, and phenylarsine oxide were purchased from Sigma Aldrich (Saint Louis, MO, USA). Soya lecithin, cholesterol, disodium hydrogen phosphate (Na_2_HPO_4_), and phosphate-buffered saline pH 7.4 (10X solution) were purchased from Fisher Scientific (Fisher BioReagents, Fair Lawn, NJ, USA). Oleic acid was provided by Croda Inc. (Edison, NJ, USA). Propylene glycol (PG) (Ekichem, Joliet, IL, USA) and sodium lauryl ether sulfate (SLES, 27% solution; Chemistry connections, Conway, AR, USA) were used. Organic solvents, such as chloroform, acetonitrile, ethanol, etc., were purchased from Pharmaco-Aaper (Brookfield, CT, USA). Dermatomed human skin was procured from a tissue bank under a protocol exempted by the Mercer University Institutional Review Board (H25-03003).

### 2.2. Formulation of 4-PBA-Loaded Emulsomes

A thin film hydration method was used to prepare the 4-PBA-loaded emulsomes [22,31]. Emulsomes were prepared using soya lecithin, cholesterol, stearic acid, and 4-PBA dissolved in chloroform using a magnetic stirrer for 1 h at 1200 RPM. This organic solution was then placed in a vacuum for approximately 2 h, forming a thin film upon complete chloroform evaporation. The thin film was rehydrated and thoroughly vortexed with 1X PBS. Emulsome solution was magnetically stirred at 1200 RPM overnight. Emulsomes were then isolated by filtration through a 0.22 µm PVDF filter. The emulsome nanoparticles were lyophilized and stored at −20 °C until further analysis. The formulation parameters explored include varying stearic acid–cholesterol ratios (1:1, 2:1, and 1:2), overall core lipid composition (cholesterol and stearic acid) (30–50%), and drug-total lipid ratios (1:1.5 and 1:2), as shown in Table 1, in order to observe effects on particle size, zeta potential, drug loading, etc. The particle that showed overall favorable characteristics, was used for further studies.

### 2.3. Characterization of Emulsomes

Thorough characterization was performed on an emulsome batched evaluation of drug loading, yield, particle size, polydispersity index (PDI), and zeta potential. The batch that depicted optimal characteristics was selected for further analysis, including Fourier-transform infrared spectroscopy (FT-IR), differential scanning calorimetry (DSC), and scanning electron microscopy (SEM), and was subject to stability studies.

#### 2.3.1. Particle Size, PDI, and Zeta Potential of Emulsomes

The particle size, PDI, and zeta potential of the emulsomes were determined with dynamic light scattering using the Malvern Zetasizer (Malvern Instruments Limited, Grovewood Road, Worcestershire, UK). Particle size and PDI were analyzed in clear cuvettes and measured in triplicate, with ten sets of measurements every run. Zeta potential was measured with a folded capillary cell in triplicate, with ten measurements every run. All samples were prepared by dispersing a small amount of lyophilized emulsomes in deionized water and analyzed in triplicate to ensure accuracy.

#### 2.3.2. Determination of Drug Loading (%) in 4-PBA-Loaded Emulsomes

To determine the amount of 4-PBA loaded into emulsome nanoparticles, a known quantity of lyophilized emulsome powder was dissolved in 1 mL of ethanol and kept for shaking for 4 h. The suspension was then centrifuged for 5 min. The supernatant was then filtered through a 0.22 µm syringe filter and analyzed using a validated HPLC method. The percentage of drug loading was calculated using the following formula:

“Drug Loading (%)=(Amount of encapsulated 4−PBA((mg))/(Total weight of emulsomes (mg))×100”

#### 2.3.3. Differential Scanning Calorimetry (DSC) for Drug–Lipid Interaction and Physical State Analysis 

Differential scanning calorimetry (DSC) was employed to investigate drug–lipid interactions and assess the physical state of 4-PBA within the emulsome matrix. Thermal analysis was performed using a Shimadzu DSC system (Shimadzu, Kyoto, Japan). Samples of pure 4-PBA, individual emulsome components (stearic acid, cholesterol, and soya lecithin), and the optimized emulsome formulation were sealed in standard aluminum pans. The pans were heated from 25 °C to 300 °C at a rate of 5 °C/min under a continuous nitrogen flow of 20 mL/min to prevent oxidative degradation. An empty aluminum pan was used as the reference. Heat flow as a function of temperature was recorded to evaluate changes in melting behavior, crystallinity, and possible molecular interactions between 4-PBA and the lipid components. The disappearance or shift of characteristic melting peaks in the emulsome thermogram, compared to those of the raw materials, was used to infer successful encapsulation and potential miscibility or compatibility between the drug and lipid excipients. The instrument was calibrated using indium as a standard.

#### 2.3.4. Scanning Electron Microscopy (SEM) for Surface Morphology

Surface morphology and physical characteristics of the lyophilized emulsomes were evaluated using a scanning electron microscope (JEOL JSM-IT700HR, Tokyo, Japan) performed at Emory University Integrated Electron Microscopy Core Facility, Atlanta, GA. To prepare samples for imaging, emulsomes were first dispersed in deionized water, and a small volume was deposited onto a silicon wafer. Imaging was performed at multiple magnifications.

#### 2.3.5. FT-IR Analysis for Drug–Excipient Compatibility

Fourier-transform infrared (FT-IR) spectroscopy in attenuated total reflection mode (Shimadzu, Kyoto, Japan) was used to assess potential interactions between 4-PBA and emulsome components (soya lecithin, cholesterol, and stearic acid). FT-IR spectra of the pure drug, individual excipients, and the final emulsome formulation were obtained as percentage transmittance against wavenumber. Shifts or changes in characteristic peaks were evaluated to identify potential drug–excipient interactions.

#### 2.3.6. Stability Studies

Stability testing was conducted for the optimized emulsome batch under two accelerated storage conditions. To ensure adequate sample quantities for comprehensive testing, multiple batches were prepared and pooled to create a master batch. The emulsomes were sealed in vials and placed within dessicators that were maintained at 40 °C ± 2 °C/75% RH ± 5% RH, depicting accelerated room temperature storage conditions, or 25 °C ± 2 °C/60% RH ± 5% RH to depict accelerated refrigerated storage according to ICH guidelines. Samples were analyzed for changes in percent drug loading (via HPLC), visual appearance (aggregation), particle size, PDI, zeta potential, and identifying structural changes with DSC and FTIR. Results were reported as mean ± standard error.

#### 2.3.7. Formulation of Emulsome Loaded with 4-PBA Embedded in Foam (F30)

The foam formulation was previously optimized by our lab, which demonstrated the decontamination efficacy of PAO from the layers of the skin. The lyophilized emulsomes were loaded into the previously optimized foam formulation (F30) shown in Table 2 and subject to characterization to ensure foam decontaminating properties were maintained. The emulsome-embedded foam formulation contained 1.5 mg of 4-PBA per milliliter.

### 2.4. In Vitro Release Studies

Vertical Franz diffusion cells with a permeation area of 0.64 cm^2^ and a 5 mL receptor chamber were utilized to perform in vitro release studies. The membrane used in the study was a cellulose-based dialysis with a molecular weight cut-off of 14,000 kDa, which was inserted between the donor and the receptor chamber. PBS pH 7.4 was used as the receptor solution, stirring constantly at 600 RPM. The receptor media was maintained at 37 °C for the duration of the study to ensure the membrane remained at 32 °C. The optimized emulsome foam formulation was chosen for testing. An amount of 200 µL of 4-PBA emulsomes (1.5 mg/mL of 4-PBA) was applied to the membrane in the donor chamber using a positive displacement pipette. Unencapsulated 4-PBA dissolved in PBS vehicle (1.5 mg/mL) was used as the control for the release study. The donor chamber was sealed with parafilm to avoid water evaporation. This test provides insight into the rate and extent of drug release under controlled conditions, as well as identifying the predominant release kinetics of the formulation. Samples were collected from the arm of the receptor chamber at predetermined time points (0, 0.25, 0.5, 1, 2, 3, 4, 5, 8, 22, 24 h) and analyzed using a validated HPLC method.

### 2.5. Skin Integrity Testing

Human skin was kept frozen at −80 °C until use. On the day of the experiment, samples were placed in sealed ziplock bags and thawed by immersion in pre-warmed (37 °C) 10 mM PBS. Thawed skin was cut into suitable sections to fit the donor chamber, and each section’s thickness was determined with a digital gauge (MTG-DX2, Checkline, Cedarhurst, NY, USA). For dermatomed human skin, thickness typically fell within 200–500 μm. Before proceeding with in vitro permeation testing (IVPT), the functional integrity of the skin barrier was confirmed by measuring electrical resistance. Electrical resistance, as previously optimized in our lab, involves skin sections that were first carefully mounted between the donor and receptor chambers of a vertical Franz diffusion cell, ensuring no air bubbles or leaks are along the interface [34,35]. The donor chamber was filled with 300 μL of 10 mM PBS (pH 7.4), and the receptor chamber contained 5 mL of the same buffer, which was kept at 37 °C in order to maintain the skin at 32 ± 2 °C, and magnetically stirred to keep the solution homogeneous. After the filled 10 mM PBS had equilibrated for 10 min, silver/silver chloride electrodes were placed in both chambers so that each electrode was fully submerged in the buffer solution. The electrodes were connected in series with a precision load resistor (R_L_ = 100 kΩ) and an arbitrary waveform generator (Agilent 33220A, Santa Clara, CA, USA, 20 MHz) that provided a low-amplitude sinusoidal voltage signal (V_O_ = 100 mV) to minimize potential damage to the tissue. A digital multimeter (Agilent Technologies, CA, USA) was used to record the voltage drop across the skin (V_S_) as well as across the entire circuit (V_O_).

Skin resistance (R_S_) was calculated using the following equation:

$$R_{S}=V_{S}\times R_{L}/(V_{O}-V_{S})$$

where R_L_ is the known load resistance, V_O_ is the total applied voltage, and Vs is the voltage drop measured specifically across the skin. The value obtained was normalized to the skin surface area in cm^2^. Only skin samples with a resistance of at least 10 kΩ cm^2^ were considered to have an intact barrier and were included in subsequent IVPT experiments [36].

### 2.6. In Vitro Permeation and Decontamination Studies Using In Vitro Models

In vitro experiments were conducted using a vertical Franz diffusion cell setup to evaluate emulsome-loaded foam formulations for three applications: sustained 4-PBA delivery, decontamination following PAO exposure, and post-decontamination skin permeation. Dermatomed human cadaver skin (200–500 μm) was prepared as described in Section 2.5. Skin sections were mounted between the donor and receptor chambers, with PBS (pH 7.4) in the receptor compartment maintained at 37 °C and stirred continuously at 600 rpm; the skin surface temperature was maintained at 32 °C. Test groups included emulsome-loaded foam, unencapsulated 4-PBA in foam, unencapsulated 4-PBA in PBS, and emulsomes suspended in PBS, all standardized to the same 4-PBA concentration (1.5 mg/mL). Depending on the study, skin was either left intact or exposed to PAO for a 30-min period before treatment. Decontamination testing involved PAO application followed by a short contact time with the test formulations and standardized rinsing; in these cases, the Franz cells served as the exposure and washing apparatus. Permeation studies involved applying the formulations directly to intact or PAO-exposed/decontaminated skin and collecting receptor fluid samples at predetermined intervals, replacing the withdrawn volume with fresh buffer. All receptor and skin extracts were analyzed for 4-PBA and/or PAO using the validated HPLC method.

#### 2.6.1. Evaluation of Sustained 4-PBA Delivery in Intact Human Skin

An IVPT study was performed to quantify and compare the permeation of 4-PBA from emulsome-loaded foam and control formulations using dermatomed human cadaver skin (200–500 μm thickness). Skin preparation and integrity assessment were performed as described above, with only samples exhibiting TEER values ≥ 10 kΩ·cm^2^ included in the study. Skin sections were mounted between the donor and receptor compartments of vertical Franz diffusion cells, ensuring complete contact with the receptor medium (PBS, pH 7.4) under continuous stirring at 600 rpm and maintained at 37 °C. An infinite dose (200 uL) of each test formulation was applied evenly to the stratum corneum surface using a positive displacement pipette. The formulations included unencapsulated 4-PBA (control), emulsome nanoparticles in PBS, and emulsome-loaded foam, each containing an equivalent 4-PBA concentration of 1.5 mg/mL. Receptor samples were collected at predetermined intervals over 24 h and replaced with fresh PBS to maintain sink conditions. Quantification of 4-PBA in receptor samples was performed using a validated HPLC method. This study provided insight into the ability of emulsome encapsulation and foam incorporation to achieve sustained delivery compared to the unencapsulated drug

#### 2.6.2. Determination of Decontamination Efficiency After PAO Exposure

The ability of emulsome-loaded foam to remove PAO from human skin was evaluated using an adapted IVPT-based protocol from previously established procedures [21]. Dermatomed human cadaver skin (200–500 μm) was prepared as described in Section 2.6, with TEER values ≥ 10 kΩ·cm^2^ required for inclusion. Mounted skin samples were first exposed to 100 μL of PAO in ethanol (100 mg/mL) for 30 min under uncovered donor conditions, mimicking in vivo conditions. Formulations tested included emulsome-loaded foam, control foam with unencapsulated 4-PBS, unencapsulated 4-PBA in PBS, and emulsomes in PBS. A control group consisting of PAO-exposed skin that received no treatment was included to serve as the untreated reference. After 30-minute exposure, 100 μL of the assigned formulation was applied to the contaminated skin for 5 minutes. Following treatment, the donor surface was wiped with sterile cotton applicators, rinsed three times with 1 mL PBS, and blotted dry. The skin was excised, minced, and extracted with 2 mL methanol on a platform shaker at 150 rpm for 4 h. Extracts were filtered (0.22 μm nylon membrane) and analyzed for PAO content using the validated HPLC method. Decontamination efficiency (%) was calculated as follows:

$$Decontamination\;Efficiency(\%)=\frac{Untreated\;Skin\;After\;Exposure-After\;Decontamination}{Untreated\;Skin\;After\;Exposure}$$

where “Untreated Skin After Exposure” is the mean PAO content in skin from the untreated control group after the 30 min of PAO exposure, and “After Decontamination” is the PAO content remaining after the 5 min treatment and rinse procedure.

#### 2.6.3. Assessment of 4-PBA Permeation in PAO-Challenged Skin

An IVPT study was conducted to assess the effect of prior PAO exposure on subsequent 4-PBA delivery from emulsome-loaded foam and control formulations. Dermatomed human cadaver skin (200–500 μm) was prepared as described in Section 2.6. Mounted skin samples were first exposed to 100 μL of PAO in ethanol (100 mg/mL) for 30 min under uncovered donor conditions to mimic in vivo conditions. Following the exposure period, each skin section underwent a 5-minute decontamination treatment using the assigned formulation, applied to the donor surface. The donor chamber was then wiped with sterile cotton applicators, rinsed three times with 1 mL PBS, and blotted dry to remove any residual PAO. Immediately after the rinse step, the same formulation applied during decontamination was reapplied to the cleaned donor surface to initiate the permeation phase. Samples were collected from the receptor arm at predetermined intervals over a 24 h period, with fresh PBS added after each withdrawal to maintain sink conditions. Quantification of 4-PBA in receptor samples was performed using the validated HPLC method. This experimental design allowed for direct evaluation of how prior PAO exposure and the decontamination process influenced subsequent 4-PBA penetration across the skin.

### 2.7. Quantitative Analysis Using High-Performance Liquid Chromatography

The concentrations of 4-PBA and PAO in all collected samples, including those from encapsulation efficiency, drug loading, in vitro release, permeation, and decontamination studies, were determined using a validated reverse-phase HPLC method developed in our lab. The system consisted of a Waters 2695 Separation Module (Waters, Milford, MA, USA) coupled to a 996 Photodiode Array UV detector, fitted with an Agilent Eclipse^®^ C18 column (150 × 4.6 mm, 5 μm particle size). The mobile phase consisted of acetonitrile and 10 mM disodium hydrogen phosphate buffer (pH adjusted to 7.0) in a 15:85 *v*/*v* ratio and was delivered under isocratic conditions. The column was maintained at 35 °C, with a flow rate of 1.2 mL/min and a fixed injection volume of 10 μL. Each run lasted 10 min. Under these conditions, the retention times were approximately 5.5 minutes for 4-PBA and 3.9 minutes for PAO.

### 2.8. In Vivo Efficacy of 4-PBA-Loaded-Emulsomes (E2) Against Lewisite Mediated Skin Injury

#### 2.8.1. Mice Exposure and Treatment Groups

In vivo studies were performed using both male and female mice (n = 6 per group) under an approved animal protocol from the IACUC (Institutional Animal Care and Use Committee) of the University of Alabama at Birmingham. The experimental procedure began with animal randomization into four groups. Group 1 received treatment with a topical vehicle control (30 µL ethanol). Group 2 was treated with PAO diluted in ethanol (100 µg/mouse in 30 µL). Group 3 received a similar placebo treatment, and Group 4 was treated with the drug formulation designated as 4-PBA E2 + NAC. Both placebo and drug treatments were applied topically at two consecutive treatment timepoints, the first at 30 min and the second at 4 h post-PAO exposure. The concentration of 4-PBA used in this study was based on the maximum loading capacity (0.26 mg emulsomes (E2) contained 150 µg 4-PBA per 100 µL foam), while NAC (15 mg/100 µL foam), as demonstrated earlier [15]. The placebo was prepared identically. The drug or placebo (100 µL) was applied topically to cover the PAO-exposed skin area (2 × 2 cm^2^ PAO-exposed area). After 24 h, all the groups were evaluated for skin injury. Draize scoring (scored for erythema, edema, and necrosis) was conducted as described previously. Skin bi-fold thickness was also recorded with a digital caliper and presented in millimeters (mm). Gross images of the exposed skin were taken before euthanasia. Skin biopsy and tissue samples from this study were used to analyze the impact of formulated drug treatment for assessing molecular and histopathological protective effects of 4-PBA E2 + NAC in this PAO-injury model of cutaneous inflammation and damage.

DermaLab Combo analysis: DermaLab Combo device from Cortex Technology (Aalborg, Denmark) was used to evaluate the in-situ skin conditions non-invasively. This device provided a digital record of skin color (redness), ultrasound images of the skin, and transepidermal water loss (TEWL). These parameters were measured to define the protective effects of 4-PBA E2 + NAC on skin conditions of live animals at 6 and 24 h following PAO exposure non-invasively.

#### 2.8.2. Histology, Immunohistochemistry, TUNEL Assay, Protein Multiplex, and Western Blot Analysis

Brief descriptions of these assays are provided here, while detailed methodologies are available in the cited references. Histological assessment was conducted on H&E-stained slide sections using a Keyence fluorescence microscope (BZ-X710, Osaka, Japan). These skin sections were evaluated for immune cell infiltration, epidermal-dermal separation microvesication (mv) formation, and epidermal/dermal cell integrity loss as described earlier [37]. Apoptosis was assessed using the TUNEL assay kit (Roche Diagnostics, Indianapolis, IN, USA), and cytokine levels were measured using the MILLIPLEX^®^ Mouse Cytokine/Chemokine kit (EMD Millipore, Burlington, MA, USA) on a MAGPIX Luminex system (Luminex, Austin, TX, USA) as described earlier [18]. Immunohistochemistry (IHC) for ATF4 was conducted following established protocols [38], involving deparaffinization, antigen retrieval, blocking, and staining with primary and secondary antibodies, followed by visualization of ATF4 (MA5-32364, Thermo Fisher Scientific, Waltham, MA, USA) stained cells using DAB substrate under a Keyence microscope. Skin lysates were prepared in RIPA lysis buffer (BioRad, Hercules, CA, USA) for Western blot analysis of protein, including ATF4 (Cell Signaling, Danvers, MA, USA 118,157), CHOP (Cell Signaling, 2895), and p-eIF2α (Cell Signaling, 3398). β-actin was used as a loading control.

### 2.9. Statistical Analysis

All statistical analyses were conducted using GraphPad Prism version 9 (GraphPad Software, San Diego, CA, USA). Data are reported as mean ± standard error (SE) from experiments with sample sizes of n = 3 or 4. One-way analysis of variance (ANOVA) was used to evaluate differences among groups, and Tukey’s multiple comparison test was applied for post hoc analysis. Statistical significance was defined as *p* < 0.05.

## 3. Results

### 3.1. Optimization and Characterization of 4-PBA-Loaded Emulsomes

All emulsome formulations were characterized for drug loading (%), particle size (nm), polydispersity index (PDI), and zeta potential (mV) (Table 3). Drug loading ranged from 10.09% (E3) to 28.25% (E1). The higher loading seen in E1 suggests that a moderate amount of cholesterol relative to stearic acid allowed more favorable accommodation of 4-PBA within the bilayer, possibly by maintaining an optimal degree of fluidity and packing. In liposomal systems, cholesterol optimizes drug encapsulation by modulating bilayer flexibility and packing density [39]. Increasing cholesterol content beyond this (e.g., moving toward a 1:1 ratio as in E2, or higher lecithin content as in E6) was associated with reduced loading. This may be due to tighter bilayer packing, which restricts space for the drug, or dilution of the drug within a larger lipid pool [40]. When the total lipid proportion in the formulation was increased toward the upper range (E9, E10), drug loading declined to ~11–13%. At these higher lipid levels, the bilayers may undergo structural rearrangements that trap less drug, despite having more total lipid present. Conversely, at lower lipid proportions, the drug-to-lipid ratio is higher, which favors entrapment efficiency. Particle sizes varied between 129.63 nm (E10) and 266.50 nm (E1). Increasing the proportion of phospholipid (lecithin) reduced particle size, likely due to enhanced stabilization of the vesicle surface, preventing aggregation and promoting formation of smaller vesicles during hydration. However, the smallest sizes were observed in formulations with very high total lipid levels (E10), which coincided with high polydispersity and poor uniformity, indicating that smaller size alone does not guarantee stability. Most formulations had PDIs below 0.5, indicating uniform populations suitable for reproducible skin delivery. The most uniform distributions were observed in E1 (0.33) and E2 (0.35). High lipid formulations (E9, E10) produced broader distributions (PDI = 1.00), suggesting instability or vesicle fusion during processing. This supports the observation that intermediate lipid compositions yield the most stable and homogeneous particles. All emulsomes carried a negative surface charge (−26.47 to −42.53 mV), attributed to the ionized headgroups of lecithin and fatty acids. Formulations with more lecithin in the lipid phase (E6) tended to have more negative values (−42.53 mV), which enhances electrostatic repulsion and minimizes aggregation. Stability potential was particularly strong in E2 and E6, both of which combined a high magnitude zeta potential with uniform particle populations. While E1 had the highest drug loading, its large particle size (~266 nm) and low preparation yield made it less favorable for topical delivery. E2, prepared with a balanced stearic acid–cholesterol composition and intermediate lipid proportion, achieved a smaller particle size (212.40 nm), moderate drug loading (17.01%), narrow distribution (PDI = 0.35), and high surface charge (−40.97 mV). These attributes, combined with better yield, made E2 the most promising candidate for further in vitro permeation and decontamination testing.

### 3.2. Thermal Behavior and Crystallinity Analysis Using DSC

**Figure 1:**
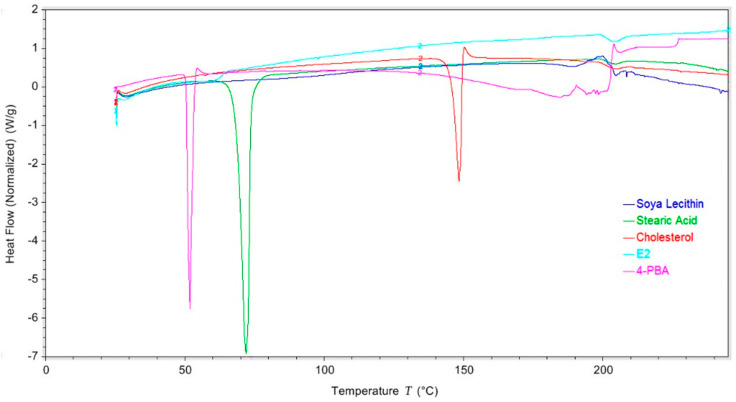
DSC thermograms of optimized emulsome and emulsome components.

The DSC thermograms (Figure 1) were used to evaluate the thermal characteristics and component interactions within the emulsome system. Pure 4-PBA displayed a well-defined endothermic peak at approximately 50 °C, confirming its crystalline nature. Stearic acid and cholesterol exhibited a sharp melting transition at 72 °C and 150 °C, respectively, with their ordered crystalline arrangements. Soya lecithin, in contrast, produced a broader thermal event at lower temperatures, which is consistent with its semi-crystalline phospholipid composition. The thermogram of the optimized 4-PBA-loaded emulsome (E2) showed no visible melting transitions corresponding to 4-PBA, stearic acid, or cholesterol. Instead, its thermal profile closely aligned with that of lecithin, indicating that the drug and solid lipid peaks were masked or eliminated due to molecular dispersion within the nanoparticle matrix. This disappearance of individual component peaks suggests that the crystalline structures of both the drug and the lipids were disrupted during nanoparticle formation. The resemblance of E2’s thermogram to lecithin supports the structural model of emulsomes, where the vesicle surface is primarily lecithin-based, and the lipid core is formed by cholesterol and stearic acid, confirming successful encapsulation of 4-PBA and proper formation of the emulsome architecture. These results collectively indicate intimate drug–lipid interactions at the molecular level, loss of crystallinity for encapsulated 4-PBA, and integration of the solid lipids into the hydrophobic core. Such structural organization is expected to contribute to the stability of the formulation and facilitate sustained release characteristics.

### 3.3. SEM Visualization of Emulsome Morphology

**Figure 2:**
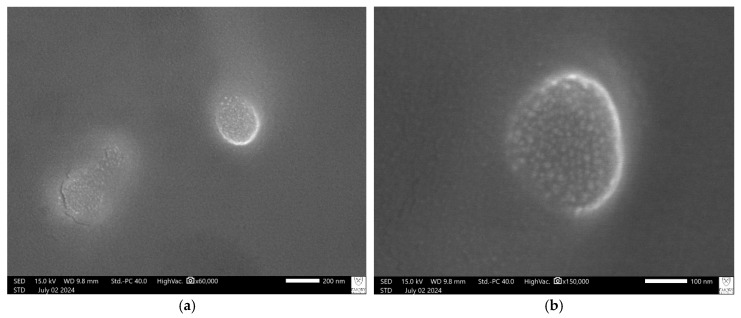
Scanning electron microscopy (SEM) images of optimized 4-PBA-loaded emulsomes (E2). (**a**) High-magnification image highlighting the surface morphology and architecture of emulsome, confirming successful vesicle formation. (**b**) Representative image of multiple emulsomes displaying uniform spherical morphology and nanometer-scale size distribution.

SEM analysis of the optimized 4-PBA-loaded emulsome formulation (E2) revealed well-formed, spherical vesicles with smooth surfaces and defined outer boundaries, confirming successful emulsome formation (Figure 2). The particles appeared uniform in morphology with consistent size distribution in the nanometer range (~100–120 nm), correlating well with DLS measurements. No signs of aggregation, surface irregularities, or structural defects were observed. The vesicles exhibited distinct, intact morphology with well-defined surface characteristics, indicating efficient assembly and structural integrity of the emulsome system.

### 3.4. Fourier-Transform Infrared Spectroscopy

**Figure 3:**
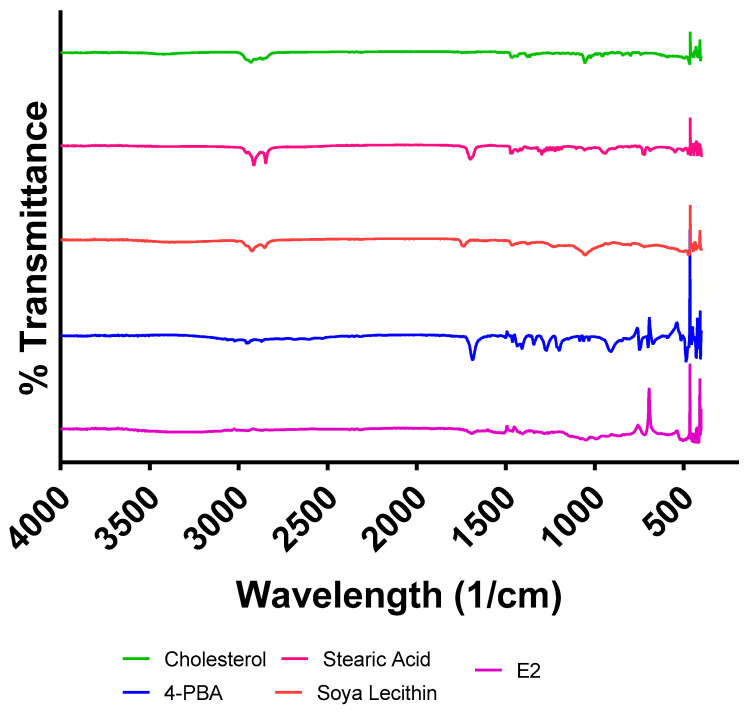
Fourier-transform infrared spectroscopy (FT-IR) analysis of cholesterol, stearic acid, soya lecithin, 4-PBA, and 4-PBA-loaded emulsome (E2).

The FTIR spectra of 4-PBA, stearic acid, cholesterol, soya lecithin, and the optimized emulsome formulation (E2) are presented in Figure 3. Each component displayed distinct vibrational bands associated with its functional groups. Pure 4-PBA exhibited key peaks at approximately 1603 cm^−1^, corresponding to aromatic C=C stretching, and multiple sharp peaks in the 700–900 cm^−1^ range attributable to out-of-plane C–H bending in the aromatic ring. These peaks confirm the presence of its conjugated aromatic carboxylic acid structure. Cholesterol showed a broad O–H stretching band at ~3400 cm^−1^, along with CH_2_ stretching vibrations at 2930–2860 cm^−1^, which is characteristic of its long aliphatic tail. Stearic acid displayed sharp CH_2_ stretching at ~2916 and ~2849 cm^−1^, and a prominent carbonyl C=O stretch near 1698 cm^−1^, which is consistent with its saturated fatty acid backbone. Soya lecithin showed a complex fingerprint region due to its phospholipid structure. Notably, a P=O stretching band was observed at ~1234 cm^−1^, and CH_2_ asymmetric and symmetric stretches appeared at ~2922 and 2852 cm^−1^, respectively. The absence of sharp carbonyl peaks near 1700 cm^−1^ suggests minimal contribution from esterified lipid domains in the free lecithin spectrum. In the emulsome formulation (E2), several changes were observed relative to individual components. A broad O–H stretching band persisted around 3390 cm^−1^, aligning with the hydroxyl groups of cholesterol and the phosphate headgroups of lecithin. Strong CH_2_ vibrations at ~2920 and 2850 cm^−1^ indicated preserved lipid hydrocarbon chain integrity. The distinct 4-PBA carbonyl band at 1687 cm^−1^ appeared highly attenuated, and the aromatic out-of-plane peaks of 4-PBA between 700–900 cm^−1^ were not detectable in the E2 spectrum, suggesting significant encapsulation of 4-PBA within the lipid matrix. This diminished intensity likely represents residual surface-associated drug. No new absorption bands were observed in E2, indicating the absence of covalent interactions or chemical degradation. Instead, the spectral overlap and disappearance of distinct drug peaks confirm that 4-PBA was physically incorporated within the emulsome core. This is further supported by the preservation of strong lecithin phosphate peaks at ~1234 cm^−1^, which is consistent with a phospholipid-rich outer shell. Overall, the spectral profile supports successful non-covalent drug entrapment, preservation of excipient structure, and the formation of a stable emulsome.

### 3.5. Stability Studies

The stability of the E2 emulsome formulation was assessed in accordance with ICH Q1A(R2) guidelines under two controlled environmental conditions. Accelerated stability testing was performed at 40 ± 2 °C and 75 ± 5% RH to simulate long-term storage stress, while intermediate refrigerated stability was evaluated at 25 ± 2 °C and 60 ± 5% RH to represent standard controlled storage conditions. Each condition was monitored in triplicate (n = 3), and samples were analyzed for one month for changes in physicochemical properties, drug content, and visual appearance. These conditions were selected to predict formulation shelf-life, identify potential degradation pathways, and ensure product quality over the intended storage period.

#### 3.5.1. Drug Loading and Physical Characterization

The short-term stability of the E2 emulsome formulation was evaluated by monitoring drug content over a 1-month period under two accelerated storage conditions as per ICH Q1A(R2) guidelines: accelerated refrigerated storage (25 °C ± 2 °C, 60% RH ± 5%) and accelerated degradation (40 °C ± 2 °C, 75% RH ± 5%) (n = 3). The initial drug loading of the formulation was 20.06% at time zero, which served as the reference point for retention analysis under both conditions. After 1 month, the emulsomes stored at 25 °C retained 19.34% drug content, equivalent to 96.4% of the original payload, indicating excellent preservation of the active compound. Under the more stringent 40 °C/75% RH condition, drug content remained virtually unchanged at 20.03%, representing a retention of 99.9%. These results demonstrate the robust encapsulation efficiency of the E2 emulsomes and their ability to maintain chemical stability even under elevated temperature and humidity for short-term storage. The minimal decline observed at 25 °C and the negligible change at 40 °C may be attributed to the tightly packed lipid bilayer structure composed of stearic acid and cholesterol, which forms a stable hydrophobic barrier around the encapsulated 4-PBA. The maintenance of drug content under high temperature and humidity further underscores the physicochemical stability of the emulsomal system, suggesting limited molecular mobility or permeability of the lipid matrix within the first month. While the slight drop in drug content at 25 °C might be indicative of initial equilibration processes, such as surface-associated drug partitioning into the external phase, the overall retention above 96% under both conditions confirms that E2 possesses strong physical integrity and encapsulation capacity. These findings highlight the potential of the formulation for short-term shelf stability and suggest that the emulsome carrier system is highly protective against premature drug leakage or degradation. However, longer-term studies are warranted to determine the threshold at which structural or compositional destabilization may occur under chronic exposure to thermal or oxidative stress.

#### 3.5.2. Emulsome Particle Size, PDI, and Zeta Potential

During the accelerated stability study, E2 demonstrated clear temperature-dependent changes in particle size, distribution uniformity, and surface charge, highlighting the impact of storage stress on colloidal integrity. At baseline, the hydrodynamic diameter was 119.4 nm, well within the favorable nanoparticle size range for skin delivery. Storage at 25 °C/60% RH resulted in an increase to 191.9 nm, whereas conditions at 40 °C/75% RH caused a more pronounced expansion to 313.4 nm, indicating early-stage vesicle fusion, lipid bilayer fluidization, or aggregation under high-temperature, high-humidity stress. The polydispersity index (PDI) values, initially 0.117 for both storage conditions, reflect a highly uniform size distribution at time zero. PDI increased to 0.365 at 25 °C and to 0.464 at 40 °C, reflecting a moderate but clear loss of homogeneity, more pronounced under accelerated room temperature conditions. These values indicate emerging heterogeneity in vesicle populations, likely due to fusion events or structural rearrangements as a result of bilayer destabilization. A PDI approaching 0.5 suggests an increased risk of physical instability, which may impact formulation reproducibility and drug release behavior. Zeta potential measurements were −34.30 ± 1.35 mV at baseline for both storage conditions. Zeta potential became more negative at 25 °C (−40.97 ± 0.81 mV), suggesting enhanced surface charge and improved colloidal stability under moderate conditions. In contrast, under 40 °C storage, zeta potential remained relatively unchanged (−35.2 ± 5.6 mV), but the increased variability and smaller magnitude of change may indicate early destabilization of electrostatic repulsion. Taken together, the data indicate that E2 retains its nanoscale size, relatively low polydispersity, and sufficient surface charge stability more effectively under refrigerated or controlled room temperature storage for at least one month. In contrast, ICH Q1A(R2) accelerated degradation conditions show early signs of compromised physicochemical stability, including larger, more heterogeneous particles with reduced surface charge magnitude. These findings support low-temperature storage as the optimal condition for preserving the short-term performance of the emulsome formulation.

#### 3.5.3. Assessment of Emulsome Stability via Differential Scanning Calorimetry

DSC thermograms were recorded to assess the physical stability of the emulsome formulation (E2) under accelerated storage conditions simulating refrigerated (25 °C, 60% RH) and room temperature (40 °C, 75% RH) environments over one month. At 25 °C, the thermal profiles remained stable, with no reappearance of the 4-PBA melting peak (~52 °C), indicating that the drug remained in a molecularly dispersed or amorphous state throughout the initial storage period. In contrast, emulsomes stored at 40 °C exhibited a modest thermal shift by Month 1, with the emergence of a broad endothermic dip beginning near 100 °C. This subtle transition may be indicative of early-stage surface-level drug rearrangement or initiation of phase separation, though no sharp melting endotherms were observed. The absence of a distinct melting peak suggests that the drug remained largely amorphous, and any incipient structural transitions were minimal. As shown in Figure S1, the thermogram at 40 °C reflects these early changes while still maintaining the broad thermal signature consistent with a stable lipid-based carrier system. These results suggest that while the formulation remains physically stable under refrigerated conditions during the first month, elevated temperature and humidity may begin to induce molecular mobility or partial lipid-drug reorganization. Continued storage under stress conditions could potentially exacerbate these changes, reinforcing the importance of optimized storage to maintain long-term integrity.

#### 3.5.4. FTIR Analysis After Stability Testing

Fourier-transform infrared spectroscopy (FTIR) was used to assess the chemical stability of the optimized emulsome formulation (E2) over a 1-month period under ICH-recommended long-term (25 °C/60% RH) and accelerated (40 °C/75% RH) storage conditions. The spectra collected at Month 0 and Month 1 demonstrated overall retention of the key structural features, with no new peak formation or major shifts in peak positions, indicating that the emulsomes maintained their physicochemical integrity during the first month of storage seen in Figure S2. Characteristic lipid-associated absorption bands including the broad O–H stretching region (~3350–3400 cm^−1^), CH_2_ asymmetric and symmetric stretching vibrations (~2920 and ~2850 cm^−1^), and phosphate P=O stretching near 1230–1240 cm^−1^ remained consistent in wavenumber and intensity, reflecting the chemical stability of lecithin, cholesterol, and stearic acid components within the vesicle structure. The carbonyl (C=O) stretching peak of 4-PBA (~1687 cm^−1^), which was masked with the emulsome due to its overlap with ester functionalities of the lipid matrix in Month 0, remained similarly obscured in the stored samples, suggesting continued encapsulation of the drug in a non-crystalline, amorphous state. Additionally, no major changes were noted in the fingerprint region (1500–1000 cm^−1^), which is sensitive to hydrocarbon backbone and drug–lipid interactions. Minor broadening was observed in the O–H and CH_2_ regions for samples stored at 40 °C, which may reflect subtle conformational shifts or hydrogen bonding alterations under thermal stress, rather than chemical degradation. These results confirm that after one month, E2 emulsomes retain their molecular architecture and drug–lipid association under both storage conditions, although minor spectral broadening at elevated temperature suggests the beginning of thermally induced lipid reorganization. Collectively, the FTIR results indicate strong chemical stability for E2 emulsomes during early storage, supporting their structural robustness for topical delivery systems.

### 3.6. In Vitro Drug Release Studies

**Figure 4:**
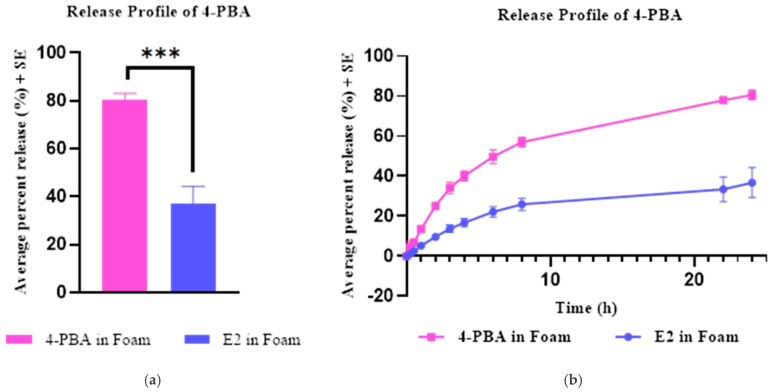
In vitro drug release study depicting the release of 4-PBA from emulsomes up to 24 h on vertical Franz diffusion cells; unpaired two-tailed *t*-test; *** (*p* ≤ 0.001). (**a**) Total receptor delivery of 4-PBA following 24-h IVRT. (**b**) Time-dependent cumulative release profile of 4-PBA demonstrating reduced release from E2 compared to free 4-PBA in foam.

The in vitro release profiles of 4-PBA from the control foam and the emulsome-loaded foam (E2) are shown in Figure 4 and summarized in Table 4. The control formulation (4-PBA in foam) exhibited a cumulative release of 78.87 ± 6.56% within 24 h, indicating a rapid liberation of drug from the foam matrix. In contrast, the E2 formulation released only 29.76 ± 4.56% over the same period, demonstrating a markedly lower overall release and effective drug retention within the emulsome structure. Kinetic modeling revealed that both formulations best fit the Higuchi model (R^2^ = 0.96 for control; R^2^ = 0.97 for E2), which is consistent with a diffusion-controlled process. Although the Higuchi release rate constant (*K*) was higher for E2 (19.78) than the control (8.96), this reflects more efficient diffusion of the fraction of drug accessible at the particle surface, while the majority remains entrapped within the lipid core and is not available for immediate release. The Korsmeyer–Peppas model also provided good fits (R^2^ = 0.96 for control; R^2^ = 0.97 for E2) with *n* values < 0.5 (0.49 and 0.46, respectively), confirming Fickian diffusion as the primary mechanism. Lower R^2^ values for the zero-order and first-order models indicate that neither a purely constant-rate nor concentration-dependent release governs the system. The Hixson–Crowell model fit was substantially better for E2 (R^2^ = 0.97) than the control (R^2^ = 0.75), suggesting that changes in particle geometry or surface area during release are more pronounced for the emulsome system, likely due to gradual lipid matrix reorganization or erosion. The simultaneous good fits to multiple models (Higuchi, Korsmeyer–Peppas, and Hixson–Crowell for E2) suggest two concurrent release mechanisms operating within the emulsome system: Fickian diffusion through the lipid bilayer matrix and surface erosion/dissolution of the lipid structure. Overall, the reduced cumulative release, combined with strong fits to diffusion-based models, supports that emulsome encapsulation prolongs the availability of 4-PBA by limiting its immediate diffusion from the foam, enabling a controlled and sustained delivery profile [41].

### 3.7. In Vitro Permeation and Decontamination Studies

#### 3.7.1. IVPT Analysis Determining Sustained Delivery of 4-PBA

**Figure 5:**
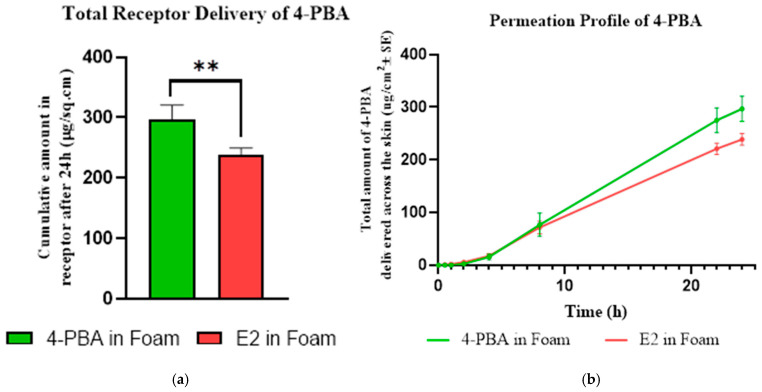
(**a**,**b**) In vitro permeation of 4-PBA from foam-based formulations over 24 h (n = 4); unpaired two-tailed *t*-test; ** (*p* ≤ 0.001). (**a**) Total receptor delivery of 4-PBA following 24-h IVPT across dermatomed human skin, comparing free drug in foam to emulsome-loaded foam (E2). (**b**) Time-dependent cumulative permeation profile of 4-PBA demonstrating reduced flux and sustained release from E2 compared to free 4-PBA in foam.

The IVPT study demonstrated distinct permeation profiles between free 4-PBA and the emulsome-loaded formulation (E2), underscoring the impact of nanoparticle encapsulation on transdermal delivery (Figure 5). Free 4-PBA in foam achieved the highest cumulative permeation after 24 h (296.80 ± 23.97 µg/cm^2^), while E2 in foam exhibited a lower permeation (238.93 ± 11.01 µg/cm^2^), confirming a reduction in drug permeation due to encapsulation. Unpaired t-test analysis revealed a statistically significant difference between the two groups ( *p* = 0.0046), with a 24.5% reduction in cumulative delivery for E2 relative to free drug. The observed decrease in drug permeation from the emulsome formulation highlights its potential for sustained release. This finding aligns with previous results from nanoparticle systems and supports the role of emulsomes as a diffusional barrier that modulates drug release kinetics. While free 4-PBA permeated rapidly across the skin, the E2 formulation provided a more gradual and prolonged release, which is consistent with the controlled diffusion expected from a lipid-based nanocarrier system. These results, when interpreted alongside complementary release modeling data, indicate that emulsomes effectively delay and sustain drug delivery across the skin barrier. The reduced variability (SD = 11.01 vs. 23.97) and tighter confidence interval also suggest a more consistent and predictable permeation pattern from E2, which is desirable for topical formulations intended for prolonged action.

#### 3.7.2. In Vitro Decontamination Efficiency of 4-PBA-Loaded Emulsome Foam

**Figure 6:**
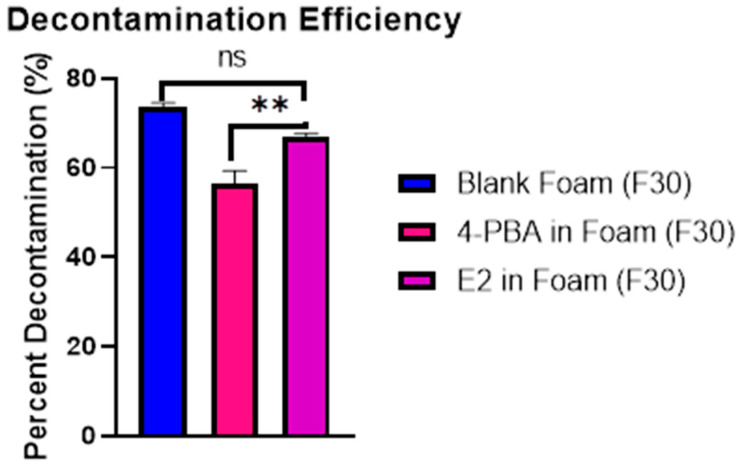
Decontamination efficiency of blank foam (F30), 4-PBA in foam (F30), and E2-loaded emulsome in foam (F30) following PAO exposure (n = 4). Data represent mean ± SD. Statistical analysis was performed using one-way ANOVA with Tukey’s post hoc test; ** *p* < 0.001; ns: not significant.

The decontamination efficiencies of the foam formulations following PAO exposure are shown in Figure 6. All formulations demonstrated effectiveness above 55%, confirming their baseline decontamination capacity. The blank foam (F30) exhibited the highest efficiency at 73.45 ± 1.27%, while the foam loaded with free 4-PBA (F30) showed a significantly reduced efficiency of 56.45 ± 1.27% (*p* < 0.0001), indicating that the free drug may interfere with the foam’s ability to remove PAO from the skin. In contrast, incorporation of the optimized emulsome formulation (E2) into the foam base resulted in a decontamination efficiency of 66.84 ± 1.27%, which was significantly higher than the free 4-PBA-loaded foam (*p* = 0.0015) but not significantly different from the blank foam (*p* > 0.05). This suggests that the emulsome formulation preserved the foam’s native cleansing efficacy, mitigating the negative impact observed with free drug loading. Together, these results support the dual utility of the E2-loaded foam: achieving therapeutic delivery without compromising its decontamination performance.

#### 3.7.3. IVPT to Assess Therapeutic Delivery of 4-PBA Following Lewisite (PAO) Exposure

**Figure 7:**
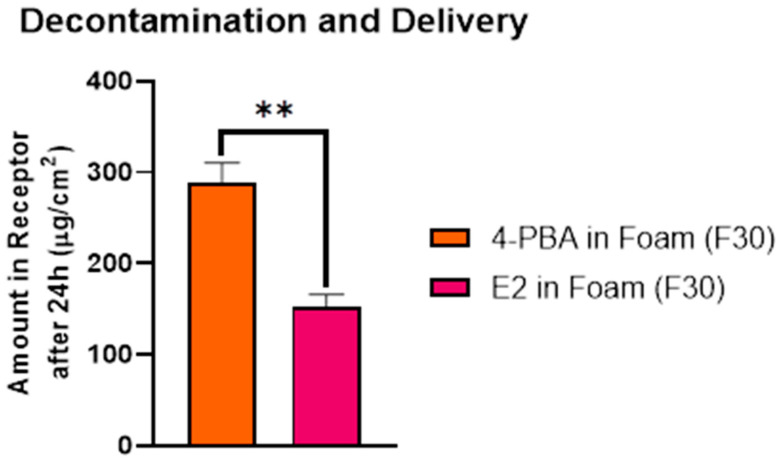
Delivery of 4-PBA into the receptor compartment following PAO exposure and foam application (n = 4). Cumulative 24-h receptor amounts are shown as mean ± SD. Statistical significance was determined using an unpaired *t*-test; ** *p* < 0.001.

To evaluate the therapeutic delivery potential of 4-PBA following chemical injury, human cadaver skin was first challenged with PAO to mimic Lewisite exposure, followed by foam-based decontamination and application of drug-loaded formulations. The cumulative 24-h delivery into the receptor compartment is illustrated in Figure 7. The formulation containing free 4-PBA in foam (F30) resulted in the highest skin permeation (288.9 ± 29.64 µg/cm^2^), whereas the E2 emulsome-loaded foam achieved significantly lower drug delivery (152.6 ± 15.29 µg/cm^2^, *p* < 0.001). This represents a 47.2% reduction in total permeation, highlighting the capacity of the emulsome carrier to modulate and control drug release even under chemically compromised skin conditions. Despite the reduced permeation, the sustained-release profile of the E2 formulation remained evident. The emulsome system effectively retained its controlled delivery characteristics, supporting its dual utility in post-exposure scenarios where both decontamination and prolonged therapeutic presence are desirable.

### 3.8. In Vivo Studies to Determine the Efficacy of 4-PBA-Loaded Emulsomes

#### 3.8.1. Foam-Formulated 4-PBA E2 + NAC Mitigates PAO-Induced Cutaneous Inflammation and Tissue Injury in Ptch1^+/−/^SKH-1 Mice

In our previous studies, we demonstrated the potential of 4-PBA, a chemical chaperon and NAC, as an antioxidant antidote against skin injury caused by exposure to arsenical vesicants [15,42]. We also demonstrated greater transdermal delivery when these drugs were dispersed in foam-based formulations. In the present work, we evaluated the efficacy of a novel topical formulation containing 4-PBA encapsulated in emulsome nanoparticles (E2) and incorporated into an F39 foam with NAC (4-PBA E2 + NAC) in a skin injury model using PAO as vesicant. PAO is a surrogate for Lewisite and has been validated as an important tool in our and others’ prior studies for assessing the efficacy of therapeutic candidates under safety conditions provided in the laboratory [37,43].

**Figure 8:**
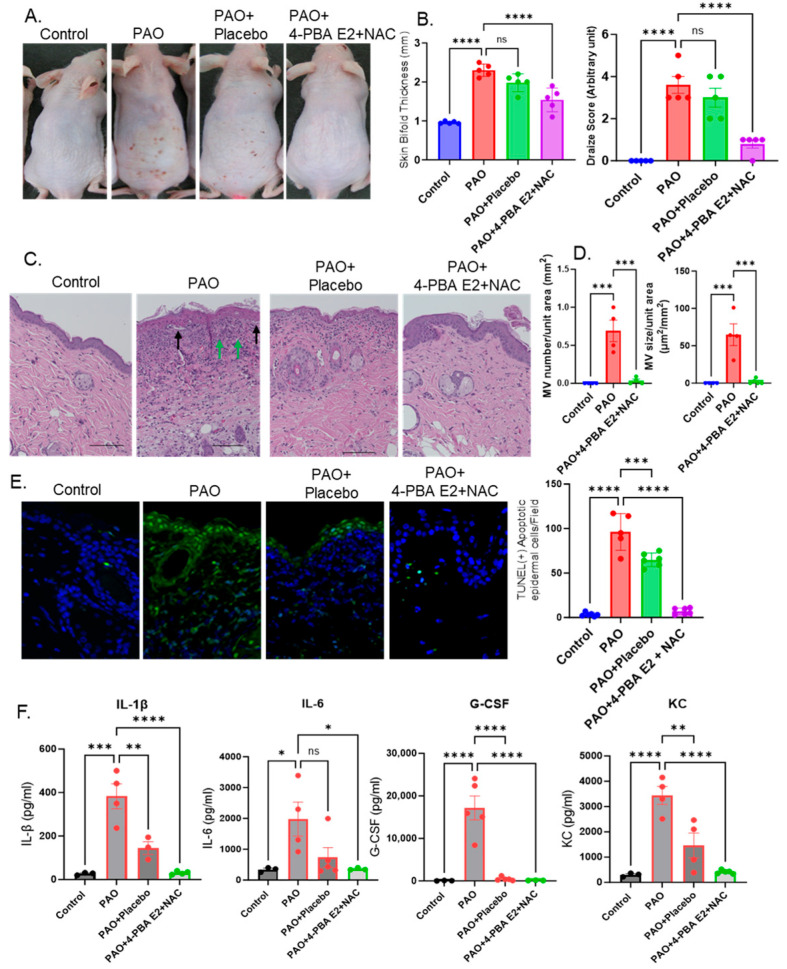
Protective effects of foam formulated “4-PBA E2 + NAC on skin damage in PAO-challenged Ptch1^+/−/^SKH-1 hairless mice. (**A**) Mice photographs showing PAO-induced cutaneous damage as observed by white wrinkly grey colored skin and pinpoint necrotic areas of exposed skin. These damaging effects were recovered by topical application of a foam formulated, 4-PBA E2 + NAC, whereas, in similar experimental conditions, placebo treatment (foam formulation without drug agents) showed some protective but non-significant effects compared to PAO-exposed skin. (**B**) Histograms showing quantitative analysis of clinical observations; calculated based on skin bifold thickness (measured by digital caliper) and Draize score (combined scoring for erythema, edema, and necrosis). (**C**) H&E staining showing histology of skin tissue; PAO-induced infiltration of inflammatory leukocytes (green arrows) and microvesicant (MV) formation (black arrows), while the foam formulated 4-PBA E2 + NAC group shows significantly reduced inflammatory cells. Scale bar 50 µm. (**D**) Microscopic MV was quantified and presented in the form of both number and size. (**E**) Microphotographs representing TUNEL-positive apoptotic epidermal and dermal cells in the skin sections obtained from the indicated treatment groups. Scale bar 50 µm. Histogram showing quantitative analysis of the green positive apoptotic cells/field. In total, 3–4 individual slides were used for analysis from each treatment group. (**F**) Quantitative analysis of protein cytokines/chemokines by multiplex. Topical treatment of foam formulated 4-PBA E2 + NAC significantly diminished the production of PAO-induced inflammatory mediators such as IL-1β, IL-6, G-CSF, and KC. *p* < 0.05 *, *p* < 0.01 **, *p* < 0.001 ***, *p* < 0.0001 **** showed significance. ns—non-significance.

Topical application of 4-PBA E2 + NAC markedly mitigated PAO-induced overall skin injury, as shown by the reduction in erythema, edema, and lesions characterized by necrotic abrasions (Figure 8A). Quantitative analysis confirmed significant reductions in the clinical observations, such as skin bi-fold thickness and Draize scores, in 4-PBA E2 + NAC-treated animals compared to PAO and PAO + placebo groups (Figure 8B). Histological examination (H&E staining) revealed extensive immune cell infiltration (green arrows) in the dermal region of the skin along with mv formation. As also described earlier, mv are microblisters characterized by the separation of epidermal and dermal layers (Figure 8C). In PAO-challenged skin, multiple mv appear, which have different sizes ranging within µm (Figure 8D). These pathological injury features were substantially diminished in the 4-PBA E2 + NAC formulation treatment. Interestingly, the placebo provided some, but insignificant, improvement (Figure 8B,C).

To assess epidermal cell apoptosis, TUNEL staining was performed in the skin sections. Under a fluorescence microscope, 4-PBA E2 + NAC treatment significantly diminished the number of apoptotic TUNEL (+) epidermal cells relative to the PAO group (Figure 8E). Placebo treatment also showed some protection against PAO-induced cell death (Figure 8E, histogram). Furthermore, multiplex cytokine analysis revealed a pronounced decrease in PAO-induced pro-inflammatory cytokines/chemokines. The protective effects were particularly apparent on the levels of IL-1β, IL-6, G-CSF, and KC, following treatment with 4-PBA E2 + NAC (Figure 8F). The placebo treatment group also showed protection against PAO-induced inflammation. Together, these findings demonstrate that topical administration of a foam-based formulation containing 4-PBA E2 + NAC significantly decreases PAO-induced skin injury, epidermal apoptosis, and inflammatory cytokine responses.

#### 3.8.2. Topical Treatment of 4-PBA E2 + NAC Improves PAO-Induced Alterations in Dermal Conditions in Mouse

**Figure 9:**
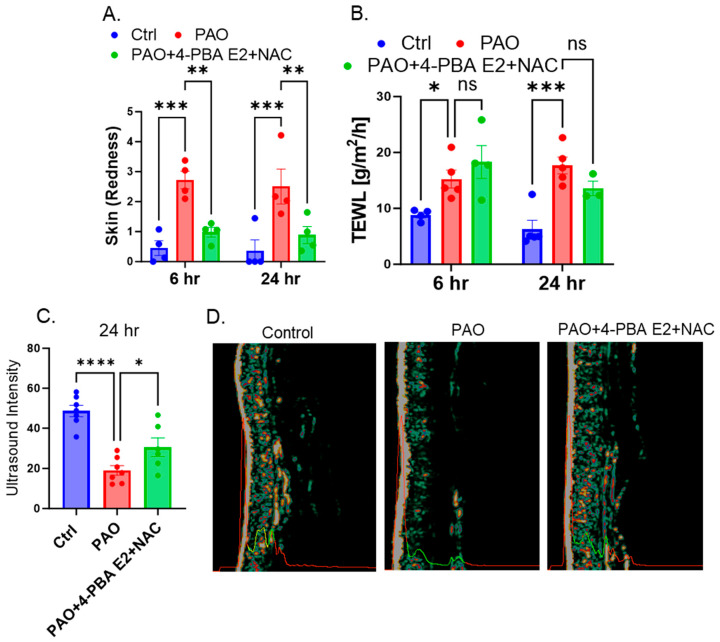
Protective effects of foam formulated ‘4-PBA E2 + NAC’ on skin conditions altered in Ptch1^+/−/^SKH-1 hairless mice challenged with PAO. DermaLab Combo device from Cortex technology (Denmark) was applied to non-invasively evaluate the skin condition, such as skin color, skin ultrasound image, and transepidermal water loss (TEWL). These parameters were measured on live animals at 6 and 24 h, following PAO exposure. (**A**) Histogram showing PAO exposure to mice’s skin significantly induced redness of the skin at both 6 and 24 h, which was significantly lowered in 4-PBA E2 + NAC-treated animals following PAO exposure. (**B**) Bar graph showing the effects of 4-PBA E2 + NAC on PAO-induced TEWL at 6 and 24 h. Topical treatment of 4-PBA E2 + NAC lowered the PAO-induced TEWL at only 24 h; however, the effect was not significant. (**C**) Histogram showing ultrasound intensity measured by the DermaLab Combo high-frequency ultrasound probe. (**D**) Ultrasonic images of mice’s dorsal skin at 24 h for the indicated treatment groups. The intensity of the color demonstrates the strength of reflected ultrasound signals. Dark color (green/black) represents low intensity while yellow bright color indicates high intensity. Compared to vehicle control, PAO-treated group represents low dermal density, while 4-PBA E2 + NAC treatment significantly rescued PAO-induced loss of dermal density. *p* < 0.05 *, *p* < 0.01 **, *p* < 0.001 ***, *p* < 0.0001 **** showed significance. ns—non-significance.

DermaLab Combo skin color probe was used to measure the redness of the skin. White calibration was performed to measure skin color before each set of measurements of individual mice. Our data showed that per cutaneous PAO exposure significantly augmented skin redness around the exposed site at both 6 and 24 h. This skin redness was significantly less following 4-PBA E2 + NAC treatment at both 6 and 24 h (Figure 9A). Next, the DermaLab Combo transepidermal water loss (TEWL) probe was used to estimate epidermal water loss from the PAO-exposed mouse skin. TEWL values were used to detect the rate of moisture loss from the damaged skin. In general, the higher value of TEWL indicates the disruption of skin barrier functions. Indeed, our data also demonstrated that compared to vehicle control, PAO exposure to mice’s skin significantly induced TEWL value at both 6 and 24 h. While post-treatment of 4-PBA E2 + NAC markedly reduced PAO-induced TEWL only at 24 h, no significant changes were observed at an early time point (Figure 9B). We also measured the dermal density of mouse dorsal skin using a high-frequency DermaLab Combo ultrasound probe. The higher the value of dermal density represents the better the skin conditions. Based on intensity, the ultrasound images demonstrate the thickness of dermis and epidermis [44]. Our data indicate that compared to vehicle control, 24 h post-PAO exposure significantly reduced mouse dermal density (Figure 9C). However, the loss of this dermal density in the PAO treatment group was compensated in animals treated with 4-PBA E2 + NAC (Figure 9C,D). Our DermaLab Combo non-invasive evaluation indicates that the formulated drug 4-PBA E2 + NAC is highly efficacious in protecting skin conditions altered by vesicant exposure.

#### 3.8.3. Foam Formulation of 4-PBA E2 with NAC Inhibits PAO-Mediated UPR Signaling in Mouse Skin

**Figure 10:**
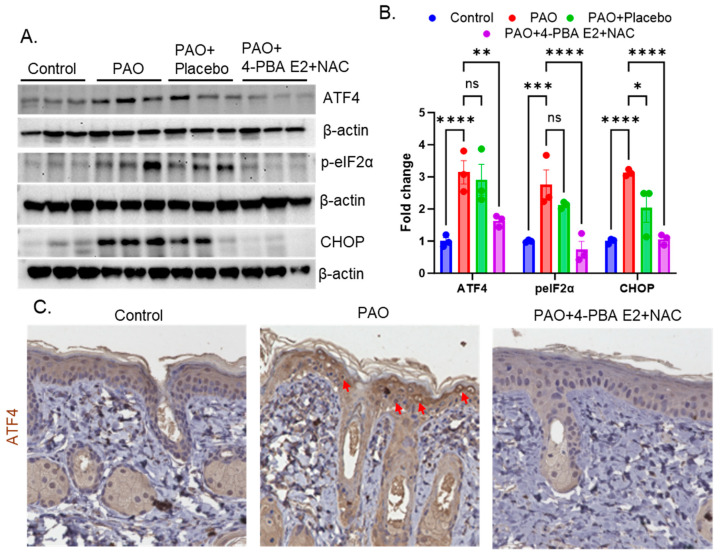
Effects of formulated ‘4-PBA E2 + NAC’ on reversal of PAO-induced unfolded protein response signaling pathway. (**A**) Expression analysis of UPR signaling proteins ATF4, p-eIF2α, and CHOP by Western blot in the skin lysates obtained from the indicated treatment groups, showing β-actin was used as an endogenous control. (**B**) Histogram showing densitometry analysis of protein bands calculated by Image J software (version 1.41o, Java 1.6.0_10) and presented in fold change. N = 3. (**C**) Immunohistochemistry of ATF4 in skin sections obtained from the indicated treatment groups. Scale bar 50 µm. Red arrows indicated nuclear translocation and enhanced expression of ATF4 in PAO-exposed skin epidermal cells. Treatment with foam formulated 4-PBA E2 + NAC significantly reduced PAO-induced expression and nuclear localization of ATF4 in skin epidermis. *p* < 0.05 *, *p* < 0.01 **, *p* < 0.001 ***, *p* < 0.0001 **** showed significance. ns—non-significance.

Earlier, we demonstrated that 4-PBA and NAC (as neat compounds) act by blocking arsenical-mediated ER stress and associated UPR signaling both in vitro and in vivo experimental settings [12,13,45]. In the present work, we confirmed that the effectiveness of the formulated 4-PBA E2 + NAC was not altered. Indeed, our data demonstrate that the formulated drugs act by blocking ER stress and UPR signaling. Treatment with 4-PBA E2 + NAC significantly reduced the PAO-induced expression of UPR signaling proteins, namely ATF4, p-eIF2α, and CHOP (Figure 10A,B), whereas placebo treatment had no such effect as observed in Western blot analysis. These findings suggest that the formulated drug exerts its protective effect by downregulating ER stress-related UPR signaling without altering the established mechanism of 4-PBA in mediating vesicant injury. This outcome was further supported by IHC analysis, which revealed strong nuclear localization of ATF4 in PAO-treated skin sections. Consistent with the Western blot results, PAO-induced expression and nuclear localization of ATF4 were markedly reduced following treatment with the formulated drug (Figure 10C).

## 4. Discussion

The rapid and progressive damage caused by Lewisite exposure—manifesting as stinging sensations within seconds and followed by erythema and vesication within hours—necessitates a treatment strategy that goes beyond just decontamination [46]. While immediate intervention is critical, the delayed progression of dermal damage underscores the need for a formulation capable of providing both decontamination and sustained therapeutic delivery. In this study, we investigated the potential of emulsomes, a hybrid nanocarrier system composed of a solid lipid core stabilized by a phospholipid shell, as vehicles for topical delivery of 4-PBA, a known chemical chaperone with anti-inflammatory and ER stress-inhibitory properties [47]. Embedding the emulsomes in a topical foam enabled dual action: removal of residual chemical agent from the skin surface and prolonged release of 4-PBA to mitigate the delayed onset of symptoms.

To engineer an effective emulsome carrier, we systematically explored the impact of three critical formulation parameters: (1) stearic acid-to-cholesterol molar ratio (1:1, 2:1, 1:2), (2) total core lipid content (30–50% *w*/*w*), and (3) drug-to-lecithin ratio (1:1–1:2). These parameters were selected due to their known influence on lipid bilayer packing, membrane rigidity, and entrapment efficiency [22,48]. Our results showed that emulsomes prepared with a 1:1 stearic acid-to-cholesterol ratio exhibited an optimal balance between particle size and drug loading. Increasing the cholesterol proportion (1:2) led to larger particle sizes and broader PDI, likely due to cholesterol-induced rigidification of the phospholipid bilayer, which interferes with self-assembly during thin-film hydration [49,50]. Total core lipid content played a nuanced role. Formulations with 40% core lipid content (E1–E3) showed slightly improved drug loading but with increased particle size and polydispersity, suggesting that beyond a threshold, higher lipid content promotes vesicle fusion and instability [51]. Drug-to-lecithin ratio was also critical. The 1:1.25 ratio (E1–E3) allowed for higher 4-PBA encapsulation (~28%) with smaller particle size and PDI, while 1:2 ratios (E8) led to significantly reduced loading (~10%) due to insufficient drug content per lipid mass. Importantly, high drug loading with acceptable size and uniformity is essential to ensure that 4-PBA is retained in sufficient amounts for prolonged release while still achieving skin penetration. Zeta potential measurements for all emulsomes ranged from −25 to −40 mV, indicating moderate electrostatic repulsion and sufficient stabilization in aqueous dispersion. This negative surface charge can be attributed to ionized phosphatidylcholine head groups and may enhance interaction with positively charged skin proteins, favoring surface adhesion and sustained retention at the site of injury [52]. These physical parameters suggest that emulsomes function as a stable depot system at the application site, resisting aggregation while gradually releasing drug payload in a controlled manner. The selected formulation, E2, possessed a desirable yield, high drug loading (17.01%), monodisperse distribution (PDI = 0.35), and moderate electrostatic repulsion (−40 mv), and it was further characterized.

FTIR analysis was performed to assess the chemical compatibility between 4-PBA and lipid excipients and to evaluate the physical state of the drug after emulsome encapsulation. The spectrum of E2 retained key lipid vibrational features characteristic of lecithin’s phosphatidylcholine headgroup. The preservation of this phosphate signal, along with lecithin-associated CH_2_ bands, supports the presence of an intact lecithin monolayer coating the emulsome surface [53]. These findings validate the chemical stability and structural integrity of both the drug and excipients during emulsome formation and are consistent with a model in which lecithin forms the outer stabilizing shell and the hydrophobic core serves as the primary drug reservoir.

DSC thermograms were used to assess the thermal behavior and physical state of the components in the optimized emulsome formulation (E2) and to evaluate potential interactions between 4-PBA and the lipid matrix. Additionally, the characteristic cholesterol peak was significantly attenuated and broadened. The disappearance or reduction in intensity of these discrete transitions suggests that the crystalline structure of 4-PBA has been disrupted and that the drug is molecularly dispersed or amorphized within the lipid matrix. Similarly, the shifts and broadening of the stearic acid and cholesterol peaks indicate miscibility and thermal integration within the emulsome system, supporting the successful encapsulation of 4-PBA within the lipid bilayer or solid-lipid core, rather than its presence as an unincorporated crystalline drug [20].

The uniformity in particle shape and surface smoothness points to effective self-assembly during the film hydration process, suggesting optimal lipid packing and minimal kinetic instability during formation. The absence of morphological defects or aggregation not only confirms successful fabrication but also implies a low interfacial energy system, which is favorable for maintaining colloidal stability during storage and application. These nanoscale structural features are especially relevant for dermal delivery, as particle integrity and surface smoothness have been correlated with enhanced skin penetration, reduced irritation, and improved drug diffusion kinetics in previous lipid-based carrier studies [54].

The emulsome formulation (E2) demonstrated minimal early signs of physicochemical instability after 1 month of storage under both accelerated refrigerated (25 °C/60% RH) and room temperature (40 °C/75% RH) conditions, with changes more pronounced at elevated temperature and humidity. This size expansion suggests temperature-dependent vesicle fusion or early-stage aggregation, particularly under accelerated room temperature conditions. The PDI also rose at 40 °C, indicating increasing heterogeneity in the vesicle population. These values, while still within an acceptable range, reflect a shift toward broader size distribution, especially under thermal stress. Zeta potential at baseline was −32.1 mV, signifying strong colloidal stability. After one month, the zeta potential became more negative at 25 °C, potentially reflecting enhanced electrostatic repulsion due to structural rearrangements further favoring refrigerated conditions. DSC thermograms supported these trends. The absence of the 4-PBA melting peak (~52 °C) at baseline and following 1-month storage at 25 °C indicates the drug remained in an amorphous state with successful encapsulation. However, under 40 °C storage, a subtle endothermic shift near 100 °C emerged, consistent with minor drug migration or surface recrystallization. No sharp melting transitions were observed, confirming the retention of the amorphous state at this early stage. These findings suggest that E2 emulsomes exhibit short-term physical and thermal stability under controlled conditions, but early destabilization processes are initiated with higher temperature conditions. While long-term stability will require further monitoring, storage at 25 °C or below appears favorable for preserving emulsome structure and sustained-release potential.

The foam formulation, previously validated for skin safety and stability, consists of anionic surfactants (SLES), ethanol, and propylene glycol [21]. These components provide dual benefits: (1) decontamination via solubilization and removal of Lewisite from the skin surface and (2) enhanced skin penetration due to ethanol’s lipid-disrupting ability and PG’s humectant properties [21]. Notably, incorporation of emulsomes into the foam did not compromise the foam itself, maintaining the vehicle’s usability and skin compatibility. This confirms that emulsome-loaded foam is functionally viable as a combined decontaminant and sustained delivery system.

The in vitro release profiles revealed that free 4-PBA in blank foam exhibited extensive release (>70%), consistent with immediate diffusion of solubilized drug. In stark contrast, the emulsome-loaded formulation exhibited controlled and sustained release: the optimized batch showed approximately 30% release over 24 h. Release modeling showed the best fit to the Korsmeyer–Peppas model (R^2^ > 0.98), indicating Fickian diffusion through a lipid matrix as the dominant release mechanism. The n-values (<0.45) support this diffusion-controlled release behavior. These findings confirm that emulsomes act as controlled release reservoirs, protecting the drug within the lipid core while enabling gradual diffusion into the surrounding medium [23,55,56]. While sustained release would continue beyond 24 h, the therapeutic window for preventing acute arsenical injury is primarily within the first 24 h post-exposure and has demonstrated efficacy within this window. From a therapeutic perspective, this sustained release is critical to counteract the biphasic nature of Lewisite injury and the extended development of cutaneous injury. While the free drug can reduce immediate symptoms, sustained presence is needed to modulate delayed inflammatory cascades and ER stress-induced apoptosis.

In vitro permeation testing (IVPT) revealed significantly reduced transdermal delivery of 4-PBA from the emulsome-loaded foam (E2) compared to free drug-loaded foam, confirming the sustained release functionality of the nanocarrier system. This attenuation is indicative of diffusional modulation by the emulsome’s lipid matrix, which acts as a reservoir system, controlling drug release across the stratum corneum [49,57]. The reduced variability in E2’s permeation data also suggests a more predictable kinetic profile, ideal for topical formulations requiring prolonged therapeutic action. Importantly, the E2 formulation retained this controlled release behavior even under barrier-compromised conditions mimicking chemical exposure. This finding is notable given that PAO exposure typically enhances dermal permeability. The preservation of sustained-release characteristics under these conditions highlights the robustness of emulsome encapsulation, offering controlled delivery even in compromised skin environments. The dual functionality of the emulsome-foam system was further supported by decontamination testing post-PAO exposure. The blank foam base (F30) demonstrated high baseline decontamination efficiency (73.45 ± 1.27%). In contrast, free 4-PBA-loaded foam (F30) showed a significant reduction in decontamination capacity (56.45 ± 1.27%, *p* < 0.0001), likely due to solubilization or interference from unencapsulated drug. However, the E2-loaded foam achieved an intermediate efficiency of 66.84 ± 1.27%, which was significantly greater than F30 (*p* = 0.0015) and not statistically different from F30 (*p* > 0.05). These results suggest that encapsulating the drug in emulsomes minimizes its interference with the foam’s cleansing ability, enabling effective detoxification while delivering a therapeutic payload. Collectively, these findings demonstrate that emulsome-based encapsulation of 4-PBA not only sustains its dermal delivery over time but also preserves decontamination performance critical for post-exposure interventions. The ability to maintain controlled release in both intact and chemically compromised skin models, along with minimal disruption to the foam’s detoxification properties, underscores the promise of this hybrid formulation for managing vesicant-induced injuries such as those caused by Lewisite, which, as stated, produces acute and delayed cutaneous injuries, including erythema and vesication within hours of exposure. While immediate decontamination is vital, the delayed pathological manifestations necessitate a sustained therapeutic strategy [15,18,20].

Earlier, we demonstrated that chemical chaperon, 4-PBA, and antioxidant, NAC, provide protection against vesicant injury caused by toxic chemical exposure [12,13]. Accordingly, we developed various formulated 4-PBA + NAC for its topical use on the damaged skin areas. Our vision in this regard was faster drug delivery and effective recovery of cutaneous lesions. With this objective, our current formulation, 4-PBA E2 + NAC, showed promise. Its application not only reduced apparent skin conditions as can be visually assessed but also restored normal homeostatic regulatory functions of the skin, such as its ability to block unnecessary epidermal water loss and maintain its cellularity as non-invasively assessed by the TEWL probe of the DermaLab Combo device. Finally, the formulated drug acted in an identical manner as the non-formulated drug in terms of attenuating the molecular pathogenesis of vesicant chemicals. This was assessed by the reduced production of inflammatory mediators’ cytokines/chemokines, growth factors, and reduction in apoptotic cell death in the epidermal and hypodermal area of the skin, as well as the mechanistic underpinning represented by the ability of the formulation to reduce the expression of proteins that lead to ER stress.

These findings demonstrate that emulsomes provide a suitable nanocarrier system for 4-PBA, enabling sustained release and reduced skin permeation compared to the free drug. The combined decontamination and delivery capability of the foam-emulsome system holds significant translational potential for emergency response to arsenical skin injuries. By maintaining drug stability, offering prolonged release, and achieving skin-reservoir effects, emulsomes embedded in foam can enhance antidote efficacy, reduce dosing frequency, and improve user compliance.

## 5. Conclusions

In this study, 4-PBA-loaded emulsomes were successfully developed and optimized to enhance dermal delivery and support post-exposure decontamination following Lewisite-induced skin injury. The formulation strategy systematically explored the impact of lipid composition, stearic acid-to-cholesterol ratio, and drug-to-lipid loading on key physicochemical parameters. The optimized formulation (E2) demonstrated nanoscale vesicle size (~200 nm), narrow size distribution (PDI < 0.35), and high drug loading (17%), while FTIR and SEM analyses confirmed drug entrapment within a stable phospholipid-stabilized vesicular system. Drug release modeling revealed non-Fickian sustained release, aligning with the emulsome’s structural features and lipid matrix properties. Notably, when embedded into a pre-validated topical foam platform, the E2-loaded foam retained decontamination efficiency statistically comparable to the blank foam (*p* > 0.05), avoiding the performance drop observed with free 4-PBA-loaded foam. In vitro permeation testing on PAO-compromised human cadaver skin showed that E2-in-foam achieved a 47.2% reduction in drug permeation compared to free 4-PBA, indicating the emulsome’s ability to modulate release and prolong dermal retention under damaged barrier conditions.

Together, these findings demonstrate the translational feasibility of emulsome-based 4-PBA delivery for arsenical skin injury. The system offers dual functionality, effective surface decontamination, and sustained therapeutic delivery, while maintaining stability, compatibility, and controlled drug presentation. This supports the continued development of emulsome-in-foam as a clinically relevant, deployable platform for emergency and post-exposure topical countermeasure applications.

## Figures and Tables

**Table 1:** Summary of 4-PBA-loaded emulsome batches.

Formulation	Stearic Acid (mg)	Cholesterol (mg)	Soya Lecithin (mg)	Drug (mg)
1	50	25	62.5	50
2	50	50	62.5	50
3	25	50	62.5	50
4	50	50	50	50
5	50	25	75	50
6	50	50	75	50
7	25	50	75	50
8	50	25	100	50
9	50	50	100	50
10	25	50	100	50

**Table 2:** Composition of previously optimized foam (F30).

FOAM (F30)	COMPOSITION (%, *v*/*v*)
OLEIC ACID	5
ETHANOL	10
SODIUM LAURYL ETHER SULFATE (27%)	20
PROPYLENE GLYCOL	65

**Table 3:** Characterization analysis of 4-PBA-loaded emulsomes.

Formulation	Stearic Acid (mg)	Cholesterol (mg)	Soya Lecithin (mg)	Drug (mg)	Drug Loading (%)	Size (nm)	PDI	Zeta Potential (mV)
E1	50	25	62.5	50	28.25 ± 0.02	266.50 ± 21.51	0.33 ± 0.02	−34.30 ± 12.36
E2	50	50	62.5	50	17.01 ± 0.00	212.40 ± 21.30	0.35 ± 0.07	−40.97 ± 1.24
E3	25	50	62.5	50	10.09 ± 0.00	265.37 ± 99.34	0.47 ± 0.08	−38.70 ± 2.19
E4	50	50	50	50	16.86 ± 0.00	207.00 ± 75.22	0.44 ± 0.02	−40.40 ± 3.12
E5	50	25	75	50	13.14 ± 0.00	235.17 ± 46.43	0.38 ± 0.04	−34.67 ± 1.78
E6	50	50	75	50	14.23 ± 0.00	195.20 ± 69.41	0.42 ± 0.03	−42.53 ± 1.01
E7	25	50	75	50	11.31 ± 0.00	147.53 ± 51.78	0.51 ± 0.16	−39.40 ± 3.86
E8	50	25	100	50	10.80 ± 0.00	225.27 ± 28.22	0.39 ± 0.03	−26.47 ± 1.42
E9	50	50	100	50	13.22 ± 0.00	186.63 ± 19.15	1.00 ± 0.16	−37.97 ± 1.53
E10	25	50	100	50	11.04 ± 0.00	129.63 ± 26.41	1.00 ± 0.03	−39.50 ± 2.85

**Table 4:** Drug release model to determine release kinetics.

Release Kinetics Model	Parameters	4-PBA in Foam	E2 in Foam
Zero-order	K	1.61	3.42
	R^2^	0.83	0.83
First order	K	−0.06	−0.06
	R^2^	0.73	0.72
Higuchi	K	8.96	19.78
	R^2^	0.96	0.97
Hixson Crowell	K	0.01	0.04
	R^2^	0.75	0.97
Korsmeyer Peppas	K	9.16	21.68
	R^2^	0.96	0.97
	n	0.49	0.46

## Data Availability

The datasets generated during and/or analyzed during the current study are included in the manuscript. Any additional data can be made available upon request.

## Supplementary Materials

The following supporting information can be downloaded at: https://www.mdpi.com/article/10.3390/pharmaceutics18010053/s1, Figure S1: DSC thermograms of emulsomes under 1 month stability analysis at accelerated refrigerated and room temperature conditions; Figure S2: FTIR spectra analyzed from wavelength 4000 to 500 cm^−1^ for E2 stored under two accelerated storage conditions: 25°C/60% RH and 40°C/75% RH.
