# Supplementary figures and images for: Emulsome-Based Nanocarrier System for Controlled 4-Phenylbutyric Acid Delivery and Mechanistic Mitigation of Arsenical-Induced Skin Injury via Foam Application
Source: Pharmaceutics. 2025 Dec 30;18(1):53. doi: 10.3390/pharmaceutics18010053 (PMC12845506; doi:10.3390/pharmaceutics18010053)

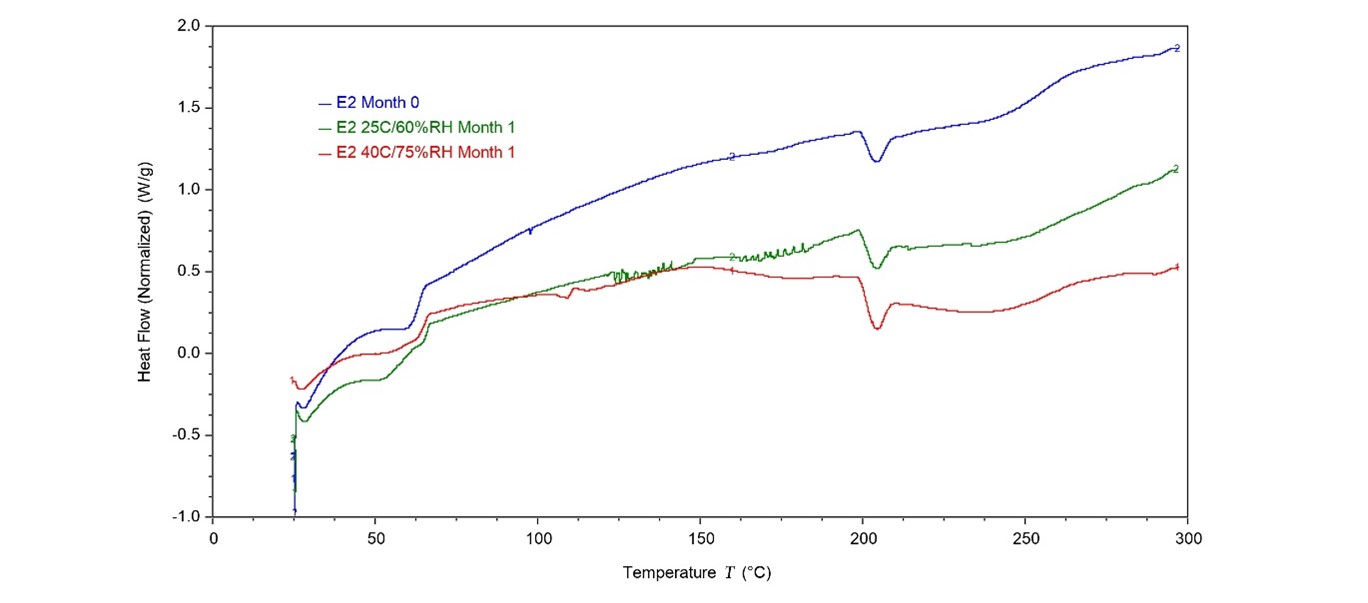

Supplement: Supplementary file 1 [file pharmaceutics-18-00053-s001.zip › Figure S1.jpg]

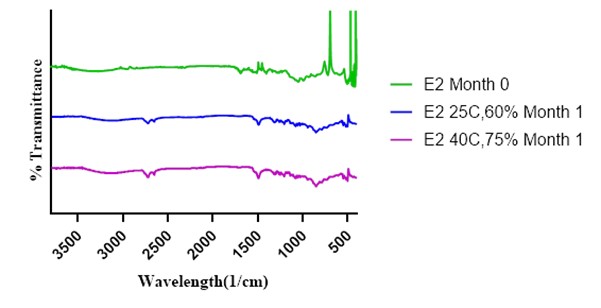

Supplement: Supplementary file 1 [file pharmaceutics-18-00053-s001.zip › Figure S2.jpg]
